# The index of tobacco treatment quality: development of a tool to assess evidence-based treatment in a national sample of drug treatment facilities

**DOI:** 10.1186/1747-597X-8-13

**Published:** 2013-03-15

**Authors:** A Paula Cupertino, Jamie J Hunt, Byron J Gajewski, Yu Jiang, Janet Marquis, Peter D Friedmann, Kimberly K Engelman, Kimber P Richter

**Affiliations:** 1 Department of Preventive Medicine and Public Health, University of Kansas Medical Center, 3901 Rainbow Boulevard, Kansas City, KS 66160, USA; 2 School of Nursing and Health Studies, University of Missouri Kansas City, 2464 Charlotte St., Kansas City, MO 64108, USA; 3 Department of Biostatistics, University of Kansas Medical Center, 3901 Rainbow Boulevard, Kansas City, KS 66160, USA; 4 University of Kansas School of Nursing, 3901 Rainbow Boulevard, Kansas City, KS 66160, USA; 5 The Schiefelbusch Institute for Life Span Studies, Dole Human Development Center, Rm 1052, 1000 Sunnyside Avenue, Lawrence, KS 66045, USA; 6 Providence VA Medical Center, 830 Chalkstone Avenue, Providence, RI 02908-4799, USA

**Keywords:** Smoking cessation, Substance abuse treatment, Tobacco use disorder, Health care services, Addiction

## Abstract

**Background:**

Quitting smoking improves health and drug use outcomes among people in treatment for substance abuse. The twofold purpose of this study is to describe tobacco treatment provision across a representative sample of U.S. facilities and to use these data to develop the brief Index of Tobacco Treatment Quality (ITTQ).

**Methods:**

We constructed survey items based on current tobacco treatment guidelines, existing surveys, expert input, and qualitative research. We administered the survey to a stratified sample of 405 facility administrators selected from all 3,800 U.S. adult outpatient facilities listed in the SAMHSA Inventory of Substance Abuse Treatment Services. We constructed the ITTQ with a subset of 7 items that have the strongest clinical evidence for smoking cessation.

**Results:**

Most facilities (87.7%) reported that a majority of their clients were asked if they smoke cigarettes. Nearly half of facilities (48.6%) reported that a majority of their smoking clients were advised to quit. Fewer (23.3%) reported that a majority of their smoking clients received tobacco treatment counseling and even fewer facilities (18.3%) reported a majority of their smoking clients were advised to use quit smoking medications. The median facility ITTQ score was 2.57 (on a scale of 1–5) and the ITTQ displayed good internal consistency (Cronbach’s alpha = .844). Moreover, the ITTQ had substantial test-retest reliability (.856), and ordinal confirmatory factor analysis found that our one-factor model for ITTQ fit the data very well with a CFI of 0.997 and an RMSEA of 0.042.

**Conclusions:**

The ITTQ is a brief and reliable tool for measuring tobacco treatment quality in substance abuse treatment facilities. Given the clear-cut room for improvement in tobacco treatment, the ITTQ could be an important tool for quality improvement by identifying service levels, facilitating goal setting, and measuring change.

## Introduction

Understanding the prevalence and quality of tobacco treatment services for drug treatment patients should be a public health priority. People with mental illness or substance abuse problems consume nearly half (44%) of all cigarettes smoked in the U.S. [1]. Drug treatment patients are interested in quitting smoking, and quitting smoking does not adversely affect short-term drug use outcomes [2-15]. Indeed, quitting smoking in the first year of drug treatment predicts better long-term substance abuse outcomes [16].

U.S. Public Health Service (PHS) tobacco treatment guidelines recommend that high quality, evidence-based care include the following: a) *all* smokers be offered treatment, b) patients *unwilling* to quit be provided with brief intervention to build motivation, and c) patients *willing* to quit be offered evidence-based treatment [17]. The highest abstinence rates are achieved when pharmacotherapy is combined with intensive counseling [18]. Office-based intervention should follow five major steps (The “*5 A*’*s*”): Ask, Advise, Assess, Assist and Arrange follow-up. The guidelines also recommend that office-based systems identify, track, and follow-up with smokers at every visit and remind providers to intervene with every smoker. Finally, substance abuse treatment facilities should adhere to treatment guidelines for the general population and should incorporate new interventions, that are effective for those in drug treatment, as new treatments become available.

To date, the prevalence and quality of tobacco treatment in drug treatment has been poorly described. In 1998 the Substance Abuse and Mental Health Service Administration’s (SAMHSA’s) Uniform Facility Data Set survey found that only 20% of U.S. substance abuse treatment facilities offered any smoking cessation services [19]. A more recent (2001) survey of Canadian drug abuse treatment facilities found that 54% “offered clients help quitting smoking,” but only 10% had any formal group or individual therapy dedicated to smoking cessation and fewer than 1% of facilities offered quit smoking medications [20]. Friedmann et al. found that somewhat more U.S. facilities provide formal counseling (38%) and pharmacotherapy (17%) [21]. Walsh and colleagues (2005) estimated that Australian substance abuse treatment programs provide brief advice to quit to 36% of clients who smoke; education about the risks of smoking to 39%, counseling to quit to 26%, and quit smoking medications to 15% [22]. Knudsen and colleagues surveyed U.S. counselors about the frequency with which they provide five brief behavioral interventions for tobacco use (0 = never, 5 = always); the interventions included assessing current tobacco use, assessing past tobacco use, advising tobacco users to quit, assessing willingness to quit, and using brief intervention to increase motivation to quit [23]. They found that counselors on average provided interventions infrequently, with a mean scale score of 2.69.

Three recent studies examined the effects of a 2008 New York State (NYS) policy change that required all publicly funded drug treatment facilities to offer tobacco dependence treatment to clients. In a survey of staff and clients from a random sample of 10 programs conducted before and one year after policy implementation, Guydish and colleagues found that client smoking prevalence diminished significantly (69.4% to 62.4%) but that implementation of tobacco treatment services differed by facility type [24]. Clients in outpatient facilities reported no pre-policy to post-policy change in the amount of tobacco treatment services they received. Those in residential treatment received less services after the change took effect. Clients in methadone treatment reported receiving more services post-implementation. Eby and colleagues surveyed 147 clinicians associated with 13 facilities immediately before and one year after the NYS policy went into effect [25]. They found that the manner in which the new policies were implemented in each facility predicted staff perceptions of how fairly the change occurred. Perceived fairness was in turn predictive of staff provision of tobacco treatment, psychological strain, and behavioral strain. Last, Brown and colleagues summarized state records and surveyed a randomly selected sample of directors of 285 facilities 3 years before and 9–12 months after policy implementation [26]. Compared to before the policy, at post-assessment a greater proportion of administrators reported they “always or formally” screened clients for tobacco use; also, they reported a greater number of tobacco services were available for clients. State discharge data on medications administered during treatment found a significant increase in the percentage of clients receiving nicotine replacement therapy, from 3.0% before implementation to 6.3% after implementation.

These studies suggest that treatment practices vary greatly, are far from routine, and are of poor quality. For example, although quit smoking medications are known to double quit rates, few clinics offer or recommend them. Moreover, no comprehensive measures of tobacco treatment quality exist – none of the studies measured all PHS guideline recommended elements of evidence-based care. Only one study assessed whether facilities provided motivational interventions to unmotivated smokers [27] and several failed to assess whether cessation medications were recommended or provided [19,23]. Moreover, the NYS studies suggest that policy change can increase implementation of treatment services but may increase strain on staff if implemented poorly.

Despite the low provision of tobacco treatment in drug treatment facilities, societal trends are creating new incentives for treating tobacco dependence in drug treatment. Staff attitudes toward nicotine dependence treatment appear to be changing; surveys conducted in 1999 and 2000 find more staff support for helping patients to quit smoking compared to surveys conducted in the 1980s and early to mid 1990s [28]. In the 1990s, changes in hospital tobacco policy, state laws, and local ordinances forced drug abuse treatment facilities to restrict indoor smoking and consider treating tobacco use [29]. Major organizations now recommend incorporating tobacco treatment into addictions treatment [30,31], and two states—New Jersey and New York—have launched major initiatives to incorporate tobacco treatment into drug treatment.

Research on tobacco treatment in drug treatment is in its infancy – the types of services offered have not been well described, no conceptual models for quality of care have been developed, and measures of services and patient outcomes are lacking. Although few programs offer formal services, many more will likely begin to treat nicotine dependence as external forces and patient demand for services increases. In the absence of clear criteria and measures of quality of care, programs may adopt services based on cost and convenience rather than efficacy [32]. It is important to develop a set of measures that capture how tobacco services are delivered, evaluate these practices, and ultimately disseminate effective practices throughout the treatment community.

The purpose of this paper is twofold: 1) to describe tobacco treatment provision across a representative sample of U.S. outpatient substance abuse treatment facilities, and 2) to develop and describe a brief index of tobacco treatment quality that assesses the extent to which facilities provide guideline-based treatment for tobacco dependence.

## Methods

### Sample

To obtain a representative sample of all current outpatient U.S. drug treatment facilities for adults, we derived our sampling frame from SAMHSA’s Inventory of Substance Abuse Treatment Services (I-SATS), a continuously updated, comprehensive listing of all known substance abuse treatment facilities in the United States (approximately 18,000 facilities) [33]. SAMHA’s Office of Applied Studies provided a download of descriptive data on all facilities in the I-SATS in 2008. These descriptive data are collected by the annual National Survey of Substance Abuse Treatment Services (N-SSATS), which recently had begun to collect data on whether facilities provide nicotine replacement therapy (NRT) [34]. There were 3,800 outpatient, adult facilities in the I-SATS sample. Based on a confidence interval of 95% and a margin of error of 5%, we calculated we should collect data from 400 facilities to accurately describe services provided by U.S. facilities [35]. Deriving our sample from the I-SATS enabled us to determine the representativeness of our final sample, because we could use descriptive data from the N-SSATS to compare our survey sample to non-participating facilities (see Measures and Results, below).

We stratified all 3,800 facilities by three characteristics that have consistently been associated with the likelihood of providing tobacco treatment services [21,29]. These three characteristics are Ownership (profit or non-profit), Nicotine Replacement Therapy (NRT) provision (yes/no) and whether or not facilities provide methadone treatment (yes/no) (Table 1). To achieve a final sample that mirrored the U.S. population of facilities, we pre-determined the number of facilities required to represent each strata within our sample (N = 400) and recruited from facilities in each cell until all strata were filled.

**Table 1:** Stratification of sample and comparison to remaining facilities

**Cell description**	**Non-participating N** = **3366 (%)**	**Survey sample N = 405 (%)**
1. NRT*, For-Profit, Methadone	12 (0.4)	3 (0.7)
2. NRT, Not For-Profit, Methadone	44 (1.3)	12 (3.0)
3. NRT, Not For-Profit, No Methadone	123 (3.7)	31 (7.77)
4. NRT, For-Profit, No Methadone	35 (1.0)	12 (3.0)
5. No NRT, For-Profit, Methadone	399 (11.9)	45 (11.1)
6. No NRT, Not For-Profit, Methadone	231 (6.9)	27 (6.7)
7. No NRT, Not For-Profit, No Methadone	1442 (42.8)	163 (40.2)
8. No NRT, For-Profit, No Methadone	1080 (32.1)	112 (27.7)

*NRT = Nicotine Replacement Therapy.

** Compared to all U.S. facilities, facilities in our sample were more likely to provide nicotine replacement, more likely to be rural, more likely to be certified by a mental health department, and less likely to be certified or licensed by a state substance abuse treatment facility.

### Recruitment

We recruited facilities and conducted surveys between November 2009 and November 2010.

Our study biostatistician (BG) stratified the facilities using data from the N-SSATS. He then randomly ordered the list of facilities within each strata and printed separate contact lists for each strata. Research assistants mailed out invitation letters to all facilities within each strata. The letter included a number for facilities to call to either a) participate in the study or b) opt out of the study. The letters also noted that a trained research assistant might call a facility at a later date to further describe the study and personally invite facilities to participate. Two weeks after letters were mailed a research assistant contacted facilities to invite them to participate, collect verbal consent and conduct surveys. Study staff called down strata lists until each strata was filled.

One person in a leadership position (clinic director, medical director, counseling supervisor, head nurse, or owner) from each facility completed the survey by phone, fax, email, or mail, according to responder preference. Participants were reimbursed $20.00 for their time.

It would have been ideal to interview multiple staff in each facility, or to collect data from facility treatment records. However, resources did not permit this intensity of data collection in enough facilities to obtain a representative sample of U.S. facilities. There are several national panel surveys that collect data from 1–2 persons at each facility. These include the National Drug Abuse Treatment System Survey (NDATSS), otherwise known as the Outpatient Drug Abuse Treatment Studies (ODATS), which collected data from a program director interview, a clinical supervisor interview, or both [36]. Also, the National Treatment Center Study [37] attempted to collect data from a program administrator and a lead clinical supervisor at each participating site. Some questions were directed at administrators, and some at clinical supervisors, so in many instances individual respondents were asked to report on the types and extent of services provided across their entire facility. Last, N-SSATS collects a single survey from facilities and provides a snapshot of the nature and extent of services provided at all U.S. facilities on one given day [38]. Hence, a number of surveys collect data from individual representatives on the type and extent of services provided in their facilities. We kept our survey narrow—focused on practices related to tobacco treatment—in order to increase the likelihood that respondents could respond reliably and accurately.

To conduct the test-retest analysis, a consecutive sample of 40 survey participants were invited to take part in a test–retest reliability sub-study of the ITTQ. Participants received an additional $20 for returning the second survey. Test-retest was conducted over a 2- to 4-week timeframe. The University of Kansas Medical Center Ethics Committee (IRB# 10979) approved all study procedures.

### Measures

To assess treatment provision we developed the Index of Tobacco Treatment Quality (ITTQ) using the following approach. First, we used as broad “domains” of treatment the 5 A’s from the PHS Guideline for Treating Tobacco Use and Dependence [39]. Based on this framework, we developed a broad list of items within each domain based on current guidelines, existing studies, and original qualitative research conducted in facilities [40,41]. We refined or discarded items that were not conceptually related to each of the 5 A’s. We shared our list of measures with 10 experts in providing tobacco treatment in drug treatment facilities, recruited from the Association for the Treatment of Tobacco Use and Dependence (ATTUD; http://www.attud.org). They rated items in terms of their importance for treating tobacco dependence and ability to discriminate between facilities that provide high versus low quality services. We refined the draft instrument based on expert advice, strength of association with smoking cessation outcomes in the general population, and other considerations such as feasibility and survey length.

For each item, survey participants were asked “*How many of your (clients/your clients who smoke) received …(name service)*.” Response categories were in the form of a Likert-type scale that was anchored to the percentage of smokers who were provided the service. These included *Almost None* (5% or fewer); *Few* (~25%); *About Half* (~50%); *Many* (~75%) and *Almost All* (90% or more). For example, as research assistants administered surveys, they would read the question, then the response categories and their associated patient percentages: “*How many of your current tobacco using clients were assessed for their readiness to stop using tobacco*? *Almost None*—*5*% *or fewer*; *About Half*—*about 50*%....”

It is important to note that we opted to ask respondents *what proportion of their current clients who smoke received each service*, instead of *how frequently the facility provided the services*. The latter is the more common method for assessing tobacco service provision—surveys often ask providers if they “routinely” provide one of a list of services. However, how respondents interpret “routinely” can be highly subjective. We opted to ask respondents to estimate the proportion of their current, smoking clients that received each service in order to guide them to anchor their response to a concrete number of clients who received services. This figure is readily verifiable through record reviews, which a future study could pursue to validate these measures. The Healthcare Effectiveness Data and Information Set (HEDIS) includes measures of the percentage of smokers who were advised to quit or to whom providers recommended medications or quit methods in the past year [42]. In order to reduce recall bias, we crafted somewhat different measures that asked about treatment of current clients, rather than clients seen in the past year. In addition, we developed new items to collect data on additional practices such as screening for smoking status, assessment of readiness to quit, and other services.

We assessed whether facilities had a specific location in their treatment files to record smoking status (*Yes*/*No*). We also used a scale to assess the intensity of tobacco treatment by assessing how often staff provide counseling to clients to help them quit using tobacco (1 = *Only when a client specifically requests treatment*; 2 = *Part of one session*; 3 = *An entire session*; 4 = *More than one session*; *5* = *Four or more sessions* /*It is integrated into all aspects of treatment*).

To describe facility representatives completing the survey, we collected gender, smoking status, and job title. Smoking status was assessed by the following item: *“What best describes your current tobacco use status?”* (*Current regular tobacco user*; *former regular tobacco user*; *never used tobacco regularly*). We used N-SSATS data, imported into our final database by unique facility-level identification numbers, to describe facility characteristics.

We selected a subset of 7 items to form the final Index of Tobacco Treatment Quality. Six items represented the PHS guideline recommended services with “A” level of evidence for efficacy in smoking cessation. The 7th item was the scale of treatment intensity. Each facility’s score on all 7 items was summed and then divided by 7 to yield a final score ranging between 1 and 5. A score of 1 represents facilities in which almost no clients received any services and 5 represents facilities in which almost all clients received all services.

### Analyses

We compared our sample to non-participating facilities in the U.S. to assess representativeness based on selected variables from the 2008 N-SSATS: geographic identifiers (urban or rural), ownership (for profit and not-for profit), affiliation (federal agency, religious organization or hospital), provision of nicotine replacement therapy, availability of opioid treatment, program for DUI/DWI/other offenders, program/group for criminal justice clients, facility size, licensure (state, mental health department, state health department, hospital authority or other state agency), and accreditation. We used descriptive statistics to display the number and percentage of facilities that reported that “many” to “almost all” of their smoking clients received each service. We used the chi-square and the Student’s t-tests to examine the significance of differences.

After selecting the 7 items that formed the final ITTQ, we calculated test–retest reliability. To do so, intraclass correlation coefficients (ICCs) were computed for the two administrations of the ITTQ. We report the ICC as a Kappa statistic for the final ITTQ and the 7 items that comprise it, using Shrout’s adjectives and cutoff values to interpret the degree of correlation: 0.00-0.20, slight; 0.21-0.40, fair; 0.41-0.60, moderate; 0.61-0.80, substantial; 0.81-1.00, almost perfect [43].

The interrelationship of ITTQ and its 7 items was analyzed by ordinal confirmatory factor analysis (CFA). Ordinal CFA is a generalization of Rasch models [44]. The ordinal CFA analysis was conducted by Lavaan package version 0.5-10, from R 2.15.2 [44]. When fitting the model, we used a one factor model and treated the response of each item as an ordinal variable. The model fit was evaluated by two statistical fit indexes: Comparative Fit Index (CFI > .90) and Root Mean Square Error of Approximation (RMSEA < .08) [45]. The composite reliability was calculated with the output obtained by ordinal confirmatory factor analysis [46]. Our interpretation of reliability follows Shrout’s guidelines (see above).

Finally, we calculated the mean, median, and standard deviation of the ITTQ across all facilities. We computed Cronbach’s alpha to assess internal consistency of the ITTQ [47]. To depict the distribution of scores we graphed the frequencies of the ITTQ scores of all facilities. To further describe the index, we summarize ITTQ scores by our original stratification of types of facilities, as described in Table 1. SPSS 18.0 was used for all data analyses. Statistical significance was set at α = 0.05 for all tests.

## Results

The 405 facilities within our eight strata had a similar distribution as those found in the overall facility population in the U.S. (Table 1). Of respondents, two-thirds were female and approximately half (51%) were current or former smokers. Responders held various roles in the program; clinic directors (59%), owner (12%), head counselor (8%), and other (21%).

### Study sample versus non-participating facilities

Study facilities were similar to U.S. facilities by most measures of comparison. Approximately half (48%) of facilities were privately owned and only 14% provided nicotine replacement therapy (Table 2). Most facilities in our sample did not offer nicotine replacement therapy nor methadone treatment. Most were not-for profit and few (10%) were located in a hospital. Two-thirds (66%) of the facilities had less than 100 clients. The average number of smokers was 75% and almost a quarter of respondents (23%) stated that their facility was mandated to provide tobacco treatment. Our sample had small but significant differences from U.S. substance abuse outpatient treatment facilities on 4 of 18 variables. Facilities in our sample were more likely to provide nicotine replacement, more likely to be rural, more likely to be certified by a mental health department, and less likely to be certified or licensed by a state substance abuse treatment facility.

**Table 2:** Comparison, survey sample versus non-participating U.S. outpatient facilities

		**Non-participating (N = 3395)**	**Survey sample (N = 405)**	**Chi-square**
1. Urban/Rural	Out of range score	0%	.5%	51.951**
	Missing	0.3%	0.2%	
	Mostly Urban = 1	32.2%	25.9%	
	2	18.7%	17.0%	
	3	29.3%	32.8%	
	4	9.7%	11.9%	
	Mostly Rural = 5	9.9%	11.6%	
2. Ownership	Private for-profit	45.3%	42.2%	8.692
Private non-profit		43.7%	48.4%	
State government		2.6%	2.0%	
Local government		5.2%	6.2%	
Tribal government		1.1%	0.5%	
Federal government		2.2%	0.7%	
3. Affiliated with religious organization		4.6%	6.6%	2.876
4. Located in hospital		9.0%	10.4%	0.893
5. Provides nicotine replacement		6.3%	14.4%	34.878**
6. Is/has an opioid treatment program		21.1%	21.6%	0.059
7. Is/has a program for DUI/DWI/other offenders		41.2%	38.9%	0.721
8. Has a specific program for criminal justice clients		32.3%	32.6%	0.013
9. Total number of clients enrolled as of 3/31/07		118.6 (2.8)	111.9 (6.0)	−1.006+
**Licensure**/**Certification**				
10. State substance abuse agency		89.4%	93.1%	5.341*
11. Mental health department		20.0%	14.6%	6.47*
12. State health department		42.1%	41.3%	0.071
13. Hospital authority		4.7%	6.3%	1.974
14. Other state agency		14.2%	12.1%	1.203
**Accreditation**				
15. JCAHO (Joint Commission)		17.7%	17.3%	0.047
16. CARF (Commission on Accred. of Rehab. Facilities)		25.1%	26.5%	0.375
17. NCQA (National Committee for Quality Assurance)		1.8%	1.7%	0.007
18. COA (Council on Accreditation)		4.0%	3.9%	0.001

* p < 0.05.

** p = 0.001.

+ Student’s t-test.

### Tobacco treatment services

Within our sample, most facilities (87.7%) reported that many/almost all of their clients were asked if they smoke cigarettes (Table 3). Nearly half (48.6%) reported that many/almost all of their smoking clients were advised to quit. Some (38.1%) facilities reported many/almost all of their smoking clients were asked if they were ready to quit. Fewer (23.3%) reported that many/almost all of their smoking clients received tobacco treatment counseling and even fewer facilities (18.3%) reported a majority of their clients were advised to use quit smoking medications to quit. Few (24.3%) facilities routinely reported many/almost all clients receive motivational counseling to help them become more motivated to quit.

**Table 3:** **Percentage of facilities in which *Many***-***Almost All *clients received services**

**Item stem: “How many of your clients…”**	**% (N)**
**were asked at intake if they smoke cigarettes**?^1^* (N = 404)	87.7 (336)
were asked at intake if they use tobacco products other than cigarettes? (N = 383)	70.6 (286)
**Item stem: “How many of your clients who smoke…”**	**% (N)**
were assessed for nicotine dependence or withdrawal using the *DSM IV* (*4*), *Fagerstrom Test*, or some other assessment? (N = 403)	33.3 (135)
**were assessed for their readiness to stop using tobacco**?^**2**^ (N = 383)	38.1 (146)
*Behavioral Treatment*	
**were advised that they should stop using tobacco**?^**3**^ (**N** = **383**)	48.6 (186)
**received individual or group counseling to help them stop using tobacco**?^**4**^ (N = 383)	23.3 (89)
**received counseling or brief intervention to help them become more motivated to quit**?^**5**^ (N = 383)	24.3 (93)
had goals for tobacco listed in their treatment plans? (N = 403)	18.5 (75)
were referred to self-help groups, such as nicotine anonymous? (N = 401)	10.7 (43)
were referred to a tobacco quitline for telephone counseling? (N = 403)	17.8 (72)
were referred to Internet or online resources for help with quitting? (N = 402)	8.9 (36)
were provided with written self-help materials about tobacco use and quitting? (N = 402)	28.7 (116)
*Pharmacotherapy*	
were referred, by staff in this facility, to an off-site provider—like a doctor or social services—to obtain quit smoking medications? (N = 402)	6.9 (28)
**were recommended to use quit smoking medications by staff in this facility**?^**6**^ (N = 383)**	18.3 (70)
were prescribed quit smoking medications by staff in this facility? (N = 402)	3.2 (13)
were provided with quit smoking medications by staff in this facility? (N = 401)	3.2 (13)

*Numbered items, in bold, are items that are included in the ITTQ.

**The 7th ITTQ item, an assessment of the intensity of tobacco treatment provided, used different response categories and is described in the text.

Most facilities (84.7%) reported they had a specific location in their treatment files to record smoking status (data not shown). Over half reported tobacco treatment was provided only when a client specifically asks for it (56%), while 15% reported it was part of one session for most clients, 3% reported that it was an entire session for most clients, 9% reported that it was provided in more than one session, and 17% reported that it was provided in four or more sessions or that it was integrated into all aspects of treatment for most clients (data not shown).

### Test-retest reliability

**Figure 1:**
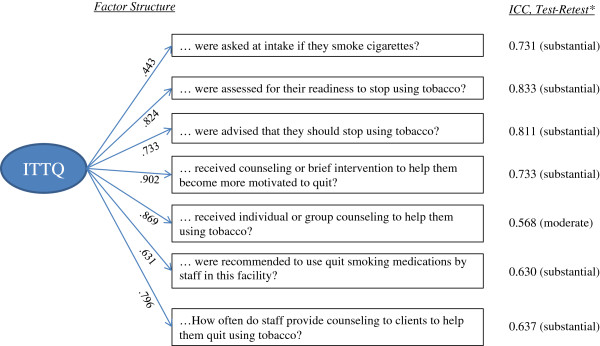
Factor structure and test-retest ICCs of seven items on ITTQ.

Figure 1 displays the ICCs for the test-retest reliability assessment of the ITTQ. The ICCs for individual index items ranging from .568 (moderate) to .833 (substantial). The test-retest ICC for the overall score of the ITTQ was .856, indicating substantial test-retest reliability (data not shown).

### Ordinal confirmatory factor analysis

The one factor model (for THE ITTQ) fits the data very well with a CFI of 0.997 and RMSEA of 0.042. The standardized loadings are also summarized in Figure 1. Item “…receiving counseling or brief intervention” has the highest loading (0.902) and is the single item most closely associated with the ITTQ domain. The calculated overall ITTQ reliability is 0.93 and average item reliability is 0.57 (not shown). Overall, the fit indexes and the composite reliability indicate the one factor ordinary CFA model fits well, and the 7 items as combined are a good summary of the ITTQ for each individual facility.

### Index of tobacco treatment quality

**Figure 2:**
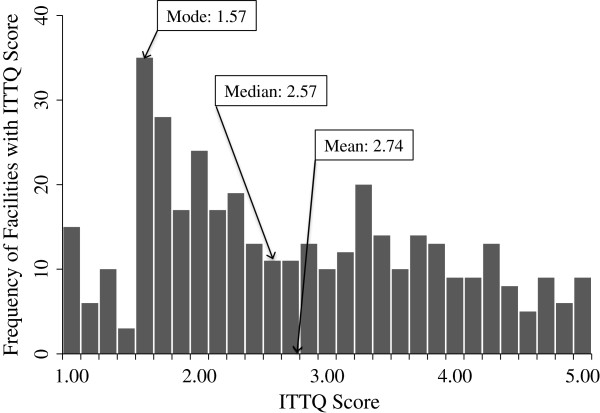
Frequencies of ITTQ Scores in 405 U.S. Substance Abuse Treatment Facilities.

The mean ITTQ score across all 405 facilities was 2.72 (Figure 2). However, the distribution of the scores was skewed to the right. For this reason, the median score (2.57) is a better summary of the overall trend in the data. Cronbach’s alpha for the ITTQ was .844, indicating good internal consistency between index items. The ITTQ score exhibited marked differences by facility type (Table 4). Nonprofit facilities that offered NRT and methadone treatment had the highest mean ITTQ score (3.98). For-profit facilities that offered neither NRT nor methadone treatment had the lowest mean ITTQ score (2.40).

**Table 4:** Mean ITTQ score by type of facility

**Cell description**	**Mean ITTQ**	**Confidence interval**
1. NRT*, For-Profit, Methadone	3.7143	(0.113, 7.316)
2. NRT, Not For-Profit, Methadone	3.9762	(3.299, 4.653)
3. NRT, Not For-Profit, No Methadone	3.6644	(3.331, 3.998)
4. NRT, For-Profit, No Methadone	3.3889	(2.783, 3.995)
5. No NRT, For-Profit, Methadone	2.4878	(2.174, 2.802)
6. No NRT, Not For-Profit, Methadone	2.6841	(2.261, 3.107)
7. No NRT, Not For-Profit, No Methadone	2.6621	(2.501, 2.824)
8. No NRT, For-Profit, No Methadone	2.3954	(2.216, 2.575)

*NRT = Nicotine Replacement Therapy.

## Discussion

The ITTQ is a brief and reliable tool for measuring tobacco treatment quality in substance abuse treatment facilities. It has very good test-retest reliability. Moreover, ordinal CFA found the ITTQ score is a good summary of each facility’s standing on 7 evidence-based treatment practices. It is sensitive to high as well as low levels of tobacco treatment, and discriminates between different types of facilities. Our development sample was representative of most U.S. drug treatment facilities, which lends confidence to the use of the ITTQ as a benchmark for tracking the prevalence and quality of treatment provision in the future. Descriptive data from the survey suggest that most facilities identify smokers but do not provide evidence-based tobacco treatment.

Findings from our overall survey accord with prior studies. The great majority of facilities routinely collect smoking status, many facilities provide brief advice to quit, some provide counseling, but few provide or recommend medications for quitting smoking [2-9,11-16,20-22,27]. It is important to note that our sample was, compared to facilities not participating in our survey, more likely to provide NRT. Although the difference was not large (6.3% versus 14.4%), it was significant and suggests that our sample may be somewhat more predisposed toward providing tobacco treatment, and the real prevalence of services is somewhat lower. The overall low prevalence of medication use in facilities may be due to the fact that many drug treatment facilities do not have prescribers on staff, counseling staff do not have the license to prescribe medications, and staff in traditionally “chemical free” facilities might be reluctant to recommend any form of medication, even over-the-counter aids.

The ITTQ could be a useful tool for quality improvement. It should, however, be subjected to validation against clinical records and patient reports to ensure it measures the true level of service provision. Moreover, it should be evaluated before and after clinical practice changes to assess its sensitivity to changes in the quantity and quality of services provided. Should it prove to be valid and sensitive, individual facilities or groups of facilities could use the ITTQ to identify their current quality score and set goals for quality improvement. Because the response categories are anchored to the number of clients receiving services, it could be converted to a chart-based survey, in which measures are derived from treatment data as opposed to self-report. This feature would be useful in settings that use paper or electronic health records [48], the latter of which could be designed to collect these measures and generate automatic reports. Because the current median ITTQ score is below the mid-range of the scale, it is likely the ITTQ will be able to detect increasing rates of treatment provision as facilities adopt new treatment practices.

Study limitations include factors common to survey research. The sample included only outpatient facilities and findings may not be applicable to inpatient facilities. Our facility sample differed significantly from non-responding clinics on 4/18 facility characteristics, which indicates our sample was somewhat different from all adult outpatient facilities. The most important of these differences was the relatively higher prevalence of NRT provision among sample facilities, which suggests that our findings may be somewhat optimistic in terms of the level of tobacco treatment. We asked one person to respond on behalf of each facility: the findings’ accuracy depends on how familiar respondents were with facility services. Respondents included only owners or top-level administrators: counselors and supervisors, as frontline workers, may have different perspectives on the extent of tobacco cessation treatment. Our surveys were self-report and hence subject to social desirability bias. To assess the validity of the ITTQ, future studies should validate it against clinical records or other forms of treatment documentation.

The study also had several strengths. Our sample size was robust. By using a sampling frame linked to facility characteristics supplied by SAMHSA, we were able to compare our sample with non-participating facilities. We constructed our survey based on treatment guidelines, existing surveys, and expert advice. Hence the survey was designed to have content validity, as it measured aspects of care supported by evidence-based treatment guidelines. Moreover, it was judged to have face validity by our team of experts. The final instrument included all recommended elements of evidence-based tobacco treatment. Response categories were anchored to numbers of clients treated and may represent closer estimates of actual provider behavior compared to prior surveys. Last, test-retest and ordinal CFA suggest the survey is reliable and a good summary score for facility performance of evidence based tobacco treatment. The ITTQ discriminated between our different strata of facilities and confirmed the findings of other studies—which suggests it is not only a brief and but also a valid measure of tobacco treatment.

## Conclusions

Offering tobacco treatment to drug treatment clients could enhance treatment outcomes and reduce long-term morbidity and mortality. Brief and valid indices such as the ITTQ are important tools for quality assessment and quality improvement. Should comparisons with treatment charts and patient reports of services further validate the ITTQ, it could serve as an outcome measure for intervention trials designed to enhance adoption of tobacco treatment in drug treatment facilities.

## Competing interests

The authors have no competing interests to declare.

## Authors’ contributions

APC helped to design the study, oversee data collection, analyze findings, and created the first draft of the report. JH supervised data collection and data entry, contributed to data analysis, and wrote sections of the manuscript. BJ contributed to survey design and supervised data analyses. YJ conducted data analyses and contributed to interpretation of the data. JM contributed to the study and the survey design, data analyses, and interpretation. PF contributed to the study and survey design and to data interpretation. KKE contributed to the study and survey design. KPR led the study and survey design, supervised JH in implementing the study, and contributed to analyses, interpretation, and drafts of the manuscript. All authors reviewed and approved drafts of the manuscript.
